# The effect of nicotine delivery system on blood protease levels: a randomized crossover study

**DOI:** 10.1038/s41598-025-19832-8

**Published:** 2025-10-14

**Authors:** Ava C. Wilson, Eleanor L. S. Leavens, Obdulia Covarrubias-Zambrano, Leah Lambart, Stefan H. Bossmann, Nicole L. Nollen, Robert Tarran

**Affiliations:** 1 Division of Genetic, Environmental, and Inhalational Disease, Department of Internal Medicine, Kansas University Medical Center, University of Kansas School of Medicine, 1034 Lied Building, Kansas City, KS 64160 USA; 2 Division of Pulmonary, Critical Care, and Sleep Medicine, Department of Internal Medicine, School of Medicine, University of Kansas, Kansas City, KS USA; 3 Department of Population Health, School of Medicine, University of Kansas, Kansas City, KS USA; 4 Department of Cancer Biology, Comprehensive Cancer Center, School of Medicine, University of Kansas, Kansas City, KS USA

**Keywords:** Biochemistry, Biomarkers, Diseases, Medical research

## Abstract

E-cigarettes and heated tobacco products are marketed as safer combustible cigarette alternatives due to their perceived potential for reduced tobacco-related toxicant exposure; however, their relative safety remains controversial. In this study we utilized serum protease levels, established biomarkers of harm contributing to lung disease, to study the effects of alternate tobacco products. Twenty-one adults who smoke cigarettes completed three visits in a randomized crossover design, separated by a 48-h washout period. Participants used their usual brand of cigarette (UBC), e-cigarette (JUUL), and heated tobacco (IQOS). We quantified serum proteases (matrix metalloproteinase (MMP) 1, MMP9, and neutrophil elastase (NE) using graphene-based nanobiosensors. UBC delivered significantly greater peak nicotine concentrations compared to JUUL or IQOS. Every device increased peak serum protease levels. After adjustment for serum nicotine, JUUL use resulted in higher levels of NE and MMP1 compared to UBC. Hierarchical clustering revealed three distinct patterns of systemic protease production that agnostically grouped by device. We demonstrated that e-cigarettes, but not IQOS, exhibited increased risk of potentially pathogenic protease release compared to UBC. These data indicate the need for prospectively designed and fully powered studies of longer duration to better understand the relative risks of e-cigarettes, IQOS and cigarettes on protease activation.

**Supplementary Information:**

The online version contains supplementary material available at 10.1038/s41598-025-19832-8.

## Introduction

It is well known that combustible cigarettes are addictive, cause multi-organ disease and lead to increased morbidity and mortality^1–3^. However, the effects of e-cigarettes (i.e. vaping) on human health have only begun to be studied. Despite this lack of long term safety data, there has been a steady uptake in e-cigarette use, and currently 9 million adults and 2.5 million adolescents use e-cigarettes in the US alone^4^. E-cigarettes are thought of as being safer than combustible tobacco because they contain fewer chemicals^5^. However, vaping causes the production of aldehydes and other reactive species, and their effects on the body are only beginning to be understood^6,7^. The American Heart Association recently concluded that “Although e-cigarettes can help some cigarette smokers quit tobacco, their appeal among adolescents has grown substantially, and limited data exist regarding their cardiopulmonary consequences”^8^. Further, several epidemiological studies have associated vaping with respiratory disease^9–12^.

Unlike traditional cigarettes, which involve the combustion of tobacco, or e-cigarettes, which vaporize an e-liquid, heated tobacco products (HTPs) operate by sufficiently heating tobacco to release nicotine-containing aerosol without burning the tobacco leaves. One of the most prominent heated tobacco products is IQOS, which electronically heats tobacco sticks to ~ 350 °C to produce a nicotine vapor. IQOS has been approved by the United States Food and Drug Administration (FDA) as a modified risk tobacco product, acknowledging its potential to reduce harm compared to cigarette smoking. Similarly, in Japan and several European nations, IQOS has gained popularity and regulatory acceptance, leading to increased adoption among smokers seeking alternatives. Despite potential benefits, the rise of heated tobacco products has also sparked debates and concerns. Public health experts and anti-tobacco advocates caution that the widespread promotion and availability of heated tobacco products could undermine efforts to reduce overall tobacco use, particularly among youth and non-smokers. As with e-cigarettes, the long-term profile of heated tobacco products is still emerging. Moreover, while heated tobacco products may constitute reduced risk, studies have demonstrated adverse effects using these products including (i) effects on human small airways^13^; (ii) the development of emphysema in naïve mice exposed to IQOS^14^; and (iii) only modest improvements in inflammation and innate defense of the lungs of mice that switched from combustible tobacco to e-cigarettes^15^.

These adverse effects may be partially driven by the activity of proteases, which are released by various cell types in response to tobacco exposure. While neutrophil elastase (NE) is exclusively released by neutrophils, matrix metalloproteinase-9 (MMP9) can be released by neutrophils, macrophages and fibroblasts, and MMP1 can be released from neutrophils, macrophages, fibroblasts and vascular cells (e.g. endothelia)^16^. Low levels of protease release are normal and are required for post-translational modification of proteins^17^. However, chronically elevated proteases are causative for significant pathology. For example, the link between elevated protease levels and lung damage is well-established: NE and MMPs degrade structural components of the lung including collagen and damage the associated endothelia^18,19^. Elevated protease levels also drive lung damage in acute respiratory distress syndrome (ARDS), CF and COPD^20^. In mice, knockdown of proteases or abolition of PMN influx into the lungs prevents cigarette-smoke-induced, protease-mediated breakdown of the lung (a precursor to emphysema)^21^. α1 anti-trypsin is a key NE inhibitor and α1 antitrypsin-deficient patients develop COPD-like lung disease at an early age due to increased NE activity^22^. Increased MMP1 levels are associated with COPD, early onset lung cancer, the release of migrating cancer cells and the formation of new tumor blood vessels^18,23,24^. Thus, proteases are validated biomarkers of harm that can be used to assess the relative safety of e-cigarettes and IQOS to combustible cigarettes. We previously reported that NE and MMP9 were significantly elevated in vapers’ bronchoalveolar lavage fluid, which suggested that vapers were at risk of chronic lung disease^25^.

Liquid biopsies have become a powerful and promising group of methods that could potentially replace most clinical biopsies performed to date^26^. They consist of the detection of biomarkers in bodily fluids of patients (serum, saliva etc.) to potentially provide information about bacterial pathogens in the lung and the immune response by the host. Graphene-based optical nanobiosensors have been established and validated in liquid biopsies for the early detection of lung cancers and ovarian cancer. They can detect protease activity in serum and feature sub-femtomolar detection limits. The objective of our study was to compare the differential effects of combustible cigarettes, e-cigarettes (JUUL), and IQOS on the production of known pathogenic protease levels. In this study, we applied novel graphene-based optical nanobiosensor technology to measure protease levels using human serum samples generated during acute use among smokers unmotivated to quit^27^.

## Methods

### Study design and ethics

**Fig. 1:**
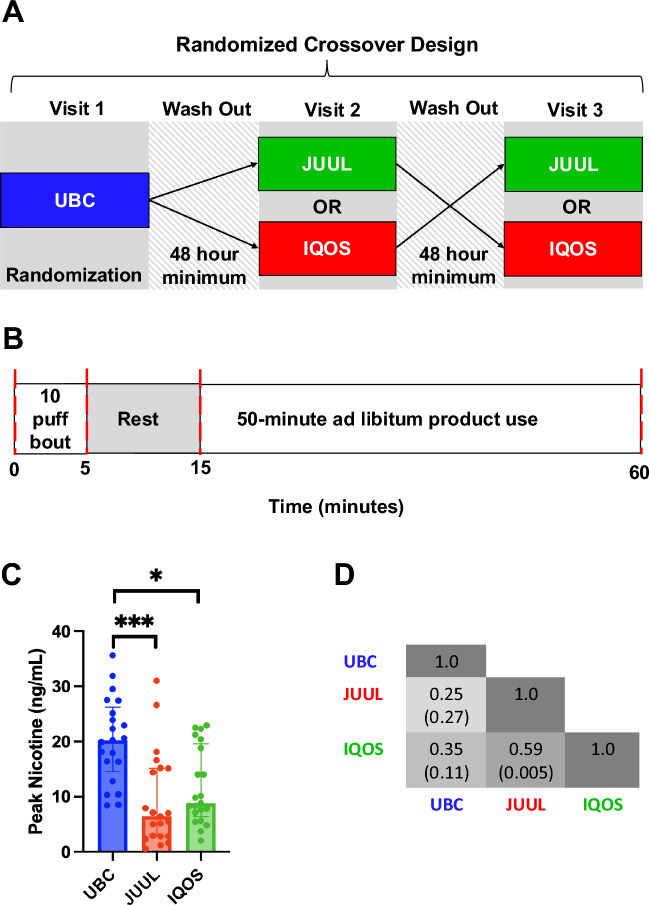
Usual brand cigarettes (UBC) yielded greater peak blood nicotine levels than JUUL and IQOS in smokers looking to try alternative products. (**A**, **B**) Study Design. (**A**) This was a randomized controlled cross-over study with three conditions: UBC, JUUL, and IQOS. At each visit, participants completed a standardized 10-puff bout followed by a 10-min rest period in which no puffs were taken followed by a 50-min ad libitum session. (**B**) Blood draws (represented as red dashed lines) occurred at 0 min (pre-10-puff bout), 5, 15 and 60 min. (**C**) Bar graph of peak serum nicotine levels by device. * = *p* < 0.05 and *** = *p* < 0.001 different as indicated. (**D**) Correlation between nicotine concentration for each device. In each cell, the correlation coefficient is represented as the top value and the *p*-value is in parenthesis.

The present study is a secondary analysis of data obtained from the “Comparative Abuse Liability Among African American and White Smokers” trial (NCT04646668). Institutional Review Board approval for the study protocol and subsequent analyses was obtained from the University of Kansas Medical Center (IRB #00,145,421). All participants in this analysis provided written informed consent prior to their inclusion. The study was designed and conducted according to the Declaration of Helsinki and ICH-GCP guidelines. The study was a randomized 3-period 2-sequence controlled cross-over design with three conditions: UBC, JUUL, and IQOS, which were separated by a period of usual behavior (Fig. 1A, B).

### Study participants

Details of the current study design have been previously described^27^, and are outlined in Fig. 1A and B. Briefly, 21 eligible participants (at least 21 years of age, smoked 5–30 cigarettes per day, smoked at their current rate for at least 6 months, not motivated to quit smoking, and had limited experience with the alternative products [used < 5 times]) were recruited from the Kansas City, MO area. Participants were provided with the study e-cigarette (JUUL in 5% nicotine) and heated tobacco (IQOS) and were given the choice between menthol or tobacco e-liquid for the e-cigarette and smooth menthol (lighter menthol flavor), fresh menthol (stronger menthol flavor), or tobacco flavor for IQOS. At each visit, following overnight abstinence, participants completed a standardized 10-puff bout followed by a 10-min rest period in which no puffs were taken, and finally, a 50-min ad libitum session (Fig. 1A and B). All participants completed 3 visits in total, which were separated by a minimum 48-h washout period. All participants used their UBC at visit 1 and then were randomly assigned to the order in which they used JUUL or IQOS at the two subsequent visits. Blood draws for serum nicotine and blood protease concentrations occurred at 0 min (pre-10-puff bout), 5 min, 15 min, and 60 min (Fig. 1B).

### Data collection and nicotine measurements

Demographic, smoking history, and nicotine dependence data were collected at baseline using self-administered questionnaires. Serum blood nicotine concentration (ng/mL) was calculated using ultra-performance liquid chromatography tandem mass spectrometry (UPLC-MSMS) and validated according to the GLP method acceptance criteria^28^. Puff topography, including puff number, was measured throughout each session via a pressure sensor attached to each device/product to capture puffing patterns.

Graphene-based nanobiosensor measurements of serum proteases.

The consensus sequences used for each protease are shown in Table 1. We have previously optimized the graphene-based nanobiosensor approach to measuring protease levels in liquid biopsies^29,30^. Briefly, a 0.03 mg/mL solution of each nanobiosensor was prepared in 25 µmol/L HEPES buffer enriched with 10 µmol/L of CaCl_2_, MgCl_2_, ZnCl_2_, pH 7.20). A total of three solutions were prepared and plated in triplicate per 96-well plate [Corning 96-Well, Flat-Bottom Polystyrene NBS Microplate Without Lid, Black]: Solution 1: Sample control (125 µL of HEPES buffer + 5 µL of collected serum). Solution 2: Assay control (125 µL of nanobiosensor solution in HEPES buffer + 5 µL of HEPES buffer). Solution 3: Assay (125 µL of nanobiosensor solution in HEPES buffer + 5 µL of collected serum). After plating, each 96-well plate was incubated at 37 °C for 1 h, and fluorescence intensities were measured using a plate reader [BioTek Synergy H1]. Fluorescence intensity was measured at λ_ex/em_: 425 ± 10 nm/650 ± 20 nm. This approach yielded a signal-to-noise ratio > 42:1.

**Table 1:** List of consensus sequences used for each protease.

Protease	Consensus sequence
MMP1	VPMS-MRGG
MMP9	VPLS-LYSG
NE	GEPL-SLLP

MMP1, Matrix Metalloproteinase 1; MMP9, Matrix Metalloproteinase 9; NE, Neutrophil Elastase.

### Statistical analyses

Descriptive statistics are presented for continuous variables as median and interquartile range (IQR) and for categorical variables as frequency and percent. Peak nicotine and protease levels were analyzed to mitigate potential type I and II errors due to multiple comparisons from multiple sampling time points. To account for the wide variation in systemic nicotine exposures across smoking devices, we normalized peak protease values to each participant’s peak serum nicotine concentration to control for differences in nicotine dose^31^. For each participant and each device, we calculated a protease-to-nicotine ratio by diving the peak measured protease level by the peak measured nicotine level. The protease/nicotine ratio was interpreted as the amount of protease generated per unit of delivered nicotine. The goal of this approach was to reduce potential confounding from nicotine itself, which can directly stimulate neutrophil protease release, allowing for a direct comparison of device-specific effects on protease biology, independent of dose differences^32^. Spearman correlation analyses were conducted to examine: (i) the relationship between individual participants’ peak nicotine concentrations across different devices; and (ii) the association between peak nicotine and peak protease levels within each device condition. Repeated measures ANOVA were used to test whether nicotine or protease levels differed by device. Pairwise post-hoc comparisons between device were performed using two-sample t-tests to identify which specific device means differed.

In addition to a 48-h minimum washout period, participants abstained from smoking > 12 h prior to the study, which was verified by measuring exhaled carbon monoxide. However, it was not known whether this duration of time was sufficient for protease activity to return to baseline for every participant, and it is possible that baseline protease levels could have been influenced by prior exposures. To account for potential confounding, we performed a sensitivity analysis using ANCOVA with baseline protease levels as a covariate to fine tune estimates of product-specific effects on protease activity. Pairwise post-hoc comparisons between device groups were performed using two-sample t-tests on the adjusted means. Hierarchical clustering was performed on peak protease levels for each device and participant using one minus the Spearman rank correlation as the distance metric (https://software.broadinstitute.org/morpheus/). Hierarchical clustering recursively merges objects based on their pair-wise distance, with objects closest together merged first and objects furthest apart merged last. What we are left with is a tree structure, or dendrogram, where leaf nodes represent the original items, and higher nodes represent the merges that occurred. All *p*-values are two-sided with *p* < 0.05 considered statistically significant. All analyses were performed using R v4.4.1.

## Results

### Characteristics of study population

Twenty-one participants were recruited for this study (Black/African American: 11; White: 10), the median age was 57 (IQR = 13) and 8 participants (38.1%) self-identified as male (Table 2).

**Table 2:** Participant demographics (n = 21) represented as Median [IQR] for continuous variables or as N (%) for categorical variables.

Age	57.0 [13.0]
% Male	8 (38.1%)
Race	
NHW	10 (47.6%)
AA	11 (52.4%)
High Nicotine Dependence	12 (60.0%)
Cigarettes Per Day	15.0 [10.0]
Smoke within 30 min. walking	17 (77.3%)
Peak Blood Nicotine Level (ng/mL)	
UBC	19.9 [12.3]
JUUL	6.4 [11.6]
IQOS	8.8 [11.2]
Peak NE Level (ng/mL)	
UBC	271.7 [72.6]
JUUL	335.1 [307.5]
IQOS	275.2 [125.5]
Peak MMP1 Level (ng/mL)	
UBC	211.3 [50.2]
JUUL	264.6 [145.5]
IQOS	223.2 [89.6]
Peak MMP9 Level (ng/mL)	
UBC	268.1 [40.1]
JUUL	293.4 [116.3]
IQOS	245.0 [78.8]

AA, African American; IQR, Interquartile Range; MMP1, Matrix Metalloproteinase 1; MMP9, Matrix Metalloproteinase 9; NE, Neutrophil Elastase; NHW, Non-Hispanic White; SD, Standard Deviation; UBC, Usual Brand of Cigarette.

### UBC use results in greater nicotine exposure compared to JUUL and IQOS

We observed significant differences in peak nicotine levels between device (F_(2,40)_ = 17.9, *p* < 0.001). Specifically, UBC delivered a significantly greater peak nicotine concentration [Median[IQR] = 20^12^ ng/mL] compared to JUUL [Median[IQR] = 6^12^ ng/mL; *t*_(20)_ =  − 5.12, *p* < 0.001] and IQOS [Median[IQR] = 9^11^ ng/mL; *t*_(20)_ =  − 4.51; *p* < 0.001] (Fig. 1C). Further, we observed a significant correlation between peak nicotine concentration across device for JUUL and IQOS, but not for UBC and either JUUL or IQOS (r_s_ = 0.59, *p* = 0.005, Fig. 1D).

### All devices significantly increased serum protease levels

**Fig. 2:**
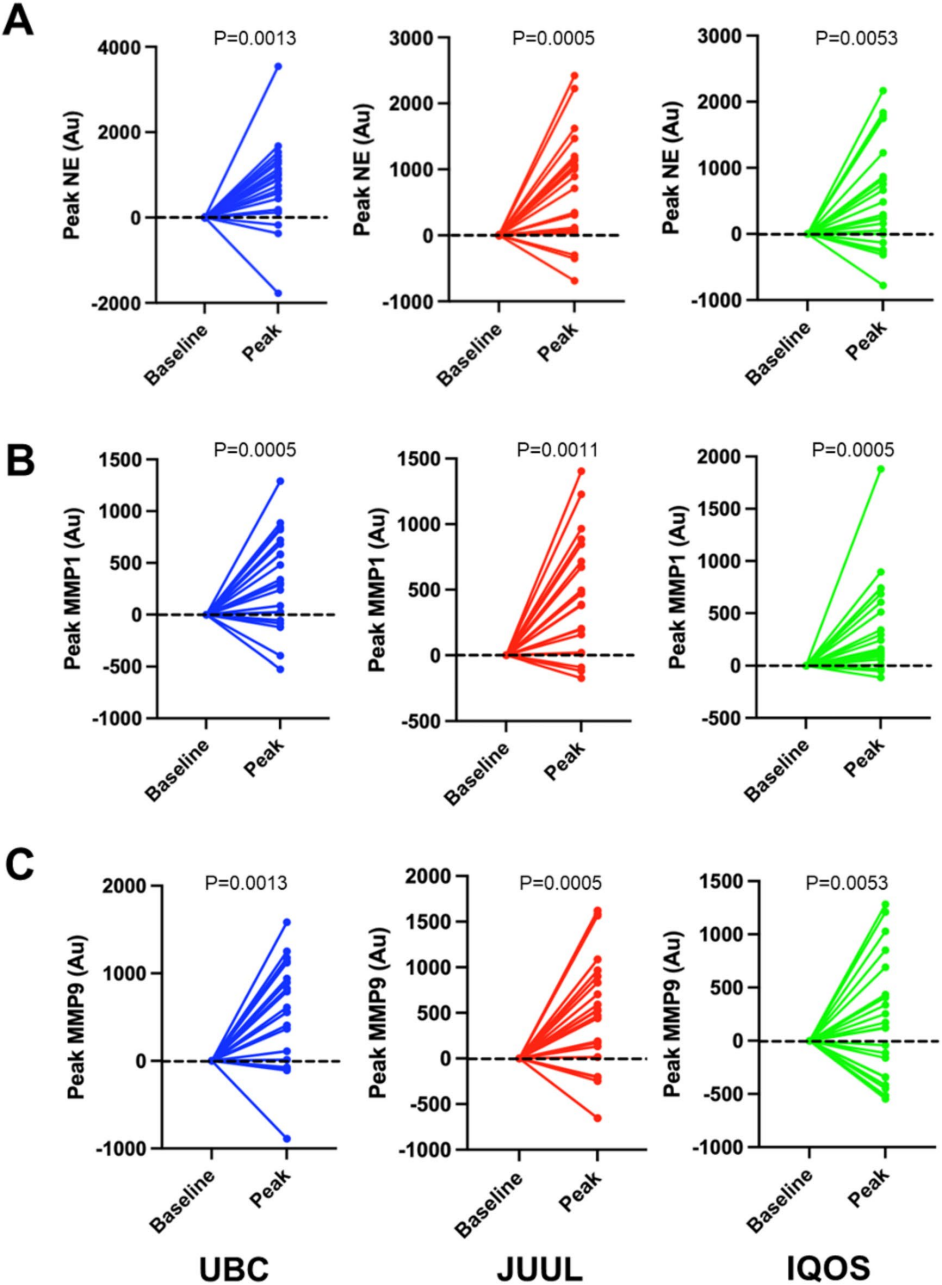
Usual brand cigarettes (UBCs), JUUL and IQOS all significantly increase serum proteases. Graphs show paired differences between baseline and peak protease level for every participant (N = 21). (**A**) Change in NE activity between baseline and peak. (**B**) Change in MMP1 activity between baseline and peak. (**C**) Change in MMP9 activity between baseline and peak. *P* values are indicated for each graph.

We measured serum protease activity in every participant using graphene-based nanobiosensors that were designed specifically for each protease (MMP1, MMP9 and NE; see Supplement Methods section). There was considerable variation in the time to peak protease activity (Fig. E1A, B, C). However, the mean time to peak protease activity was not significantly different across devices or proteases (Fig. E1D). Accordingly, we evaluated peak protease activity going forward. We did not observe any difference in protease activity based on race or gender (Fig. E2A, B). However, for all three proteases, we observed significantly increased serum protease levels from baseline to peak across every device tested (Fig. 2A, B, C). We then plotted peak protease activity by age (Fig. E1D). We acknowledge that since most participants tended to be ~ 50 years of age, there is insufficient spread to robustly consider age as a covariate. However, for NE and MMP1, JUUL use but not UBC or IQOS significantly correlated with age.

**Fig. 3:**
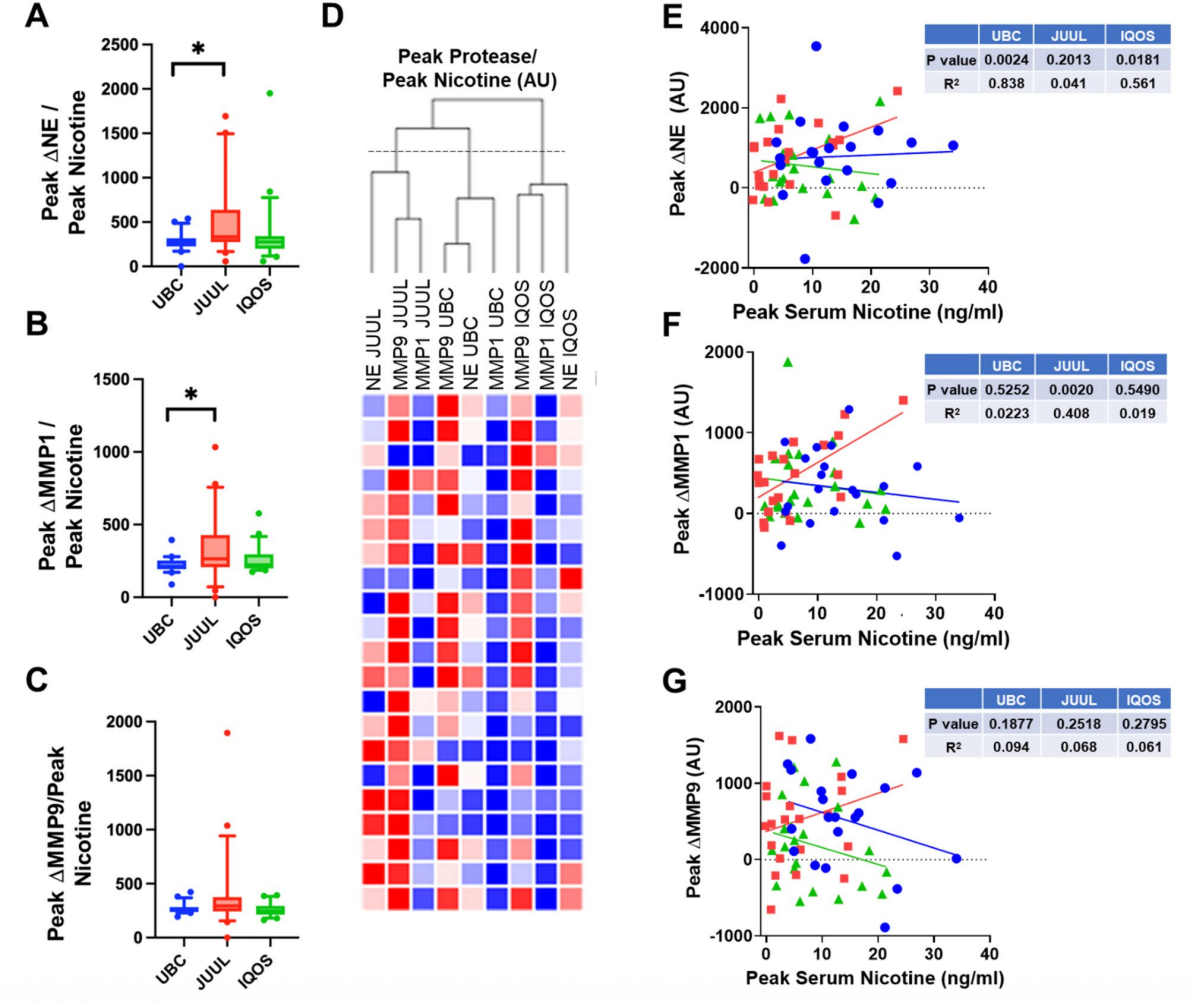
JUUL exposure results in significantly greater peak nicotine-adjusted protease activities than UBC. (**A–C**) Box plots of peak activity normalized to peak serum nicotine for NE, MMP1 and MMP9 respectively, stratified by device type. All n = 21 paired subjects. * = *p* < 0.05 different as indicated. (**D–F**) Scatter plots of peak protease activity vs peak serum nicotine levels for NE, MMP1 and MMP9 respectively, stratified by device type. All n = 21 paired subjects. (**G**) Hierarchical clustering using one minus the Spearman rank correlation as the distance metric for peak protease levels by each device for every participant (n = 21). Darker red shading corresponds to a larger, positive correlation coefficient and darker blue shading corresponds to a larger, negative correlation coefficient. The dashed line represents the tree height cut point in which three distinct clusters emerge.

Since UBC use resulted in significantly greater serum nicotine levels than the other devices (Fig. 1C), we normalized peak protease levels to peak serum nicotine to account for the differences in exposures. After normalization, JUUL use resulted in significantly higher levels of NE (Median[IQR]_JUUL_ = 335[307] AU; Median[IQR]_UBC_ = 272[73] AU; *t*_(20)_ = 2.38; *p* = 0.027) and MMP1 (Median[IQR]_JUUL_ = 265[146] AU; Median[IQR]_UBC_ = 211^50^ AU; *t*_(20)_ = 2.11, *p* = 0.048) compared to UBC (Fig. 3A, B). These findings remained statistically significant following sensitivity analyses controlling for baseline protease levels using ANCOVA. In contrast, MMP9 production was not different across groups (Median[IQR]_JUUL_ = 293[116] AU; Median[IQR]_UBC_ = 268^40^ AU; *t*_(20)_ = 1.61, *p* = 0.12; Fig. 3C). Further, IQOS protease levels were not significantly different from the other groups (Fig. 3A, B, C). We then performed hierarchical clustering where each protease was agnostically grouped. This revealed three distinct patterns, where proteases were grouped by the device that generated their measurement (Fig. 3D). Finally, we looked for correlations between protease levels and measures of exposure. We did not see a correlation between serum protease levels and puff number across all devices (Fig. E4). Interestingly, for NE and MMP1, peak protease levels significantly correlated with peak nicotine levels after Juul use (Fig. 3E, F). In contrast, in the same subjects, peak protease levels did not correlate with nicotine after UBC or IQOS. However, MMP9 levels did not correlate with serum nicotine for any device (Fig. 3G).

## Discussion

In this study, we measured nicotine and protease levels in serum of chronic combustible cigarette smokers who were unmotivated to quit cigarette smoking. We observed an increase in nicotine exposure across all devices, with UBC achieving the greatest peak nicotine levels. Since proteases are causative for several smoking-related diseases, we used them as a biomarker of harm following exposure to UBC, e-cigarettes and IQOS. Consistent with our previous studies of UBC and e-cigarette-exposed subjects, who displayed significantly increased lung proteases, including NE and MMP9, we found that UBC and e-cigarettes both significantly increased NE, MMP1 and MMP9. Previous studies of proteases in vapers vs smokers were cross-sectional in nature^25,33,34^. To the best of our knowledge, this is the first lab-based study to demonstrate that e-cigarettes can directly increase protease levels. However, previous studies have demonstrated that vaping acutely and chronically affects blood vessels. For example, flow-mediated dilation (FMD) was similarly reduced in chronic vapers and smokers, which was accompanied by a reduction in secretion of the vasodilator NO in both vapers and smokers^35^.

After adjustment for nicotine, we found IQOS induced lower levels of all 3 proteases compared to JUUL and lower levels of MMP9 compared to UBC, albeit these comparisons did not reach statistical significance. It has previously been reported that IQOS reduced exposure to known combustible cigarette smoke toxicants including lead, mercury, formaldehyde, and styrene, amongst others^36^. However, animal and cellular studies have demonstrated an association between IQOS and pulmonary, cardiovascular, and systemic toxicity^37^. Human studies comparing biomarkers of exposure and effect between IQOS and combustible cigarettes have yielded mixed results^38–43^. Interestingly, the majority of studies that reported significantly lower exposure to biomarkers of exposure and effect in HTP/IQOS users versus combustible cigarette users were funded by Philip Morris International (PMI), the world’s leading tobacco company and the manufacturer of IQOS^38,41,42,44^. An in-depth analysis of the human in vivo data collected by PMI found no statistically detectable difference between IQOS and combustible cigarette users for 33 of the 37 biomarkers of exposure and effect claimed to be reduced in IQOS users^45^. In fact, one study concluded that 95% of manuscripts without tobacco-industry conflicts of interest have reported potentially harmful effects^47^. Multiple studies have found either no evidence for harm reduction or even an increase in risk factors for adverse respiratory or cardiovascular events in IQOS compared to cigarettes and nicotine-free e-cigarettes^48–51^. Alarmingly, IQOS has been associated with acute increases in resistance and reactance within the small airways, increased airway obstruction, and increased heart rate and blood pressure that exceed the acute effects of cigarettes^48–51^. A longitudinal study conducted by Harada et al. found participants who switched from cigarettes to HTP-only use had patterns of lung function decline similar to cigarette-only smokers^52^. Additional studies have found either no difference or increased markers of risk in HTP users for metabolic syndrome, conditions related to the nervous and reproductive systems, and dental disease^53–56^. Under baseline conditions, UBC, JUUL and IQOS induced identical increases in serum protease levels (Fig. 2). Further, we observed significantly correlated peak nicotine concentrations between JUUL and IQOS (Fig. 1D). This may indicate that despite differences in formulation, both devices can achieve comparable systemic nicotine delivery. This relationship likely reflects consistent individual factors such as puffing behavior and nicotine metabolism, indicating that users who absorb nicotine efficiently on one device tend to do so on the other. Consequently, differences in protease response between JUUL and IQOS are less likely attributable to nicotine dose alone and more plausibly reflect the influence of non-nicotine constituents or differences in delivery kinetics. Taken together, these data provide initial evidence that both e-cigarettes and HTPs have negative impacts on serum protease levels. However, longer-term studies in larger samples will be required to confirm or refute these findings.

Interestingly, when normalized to nicotine, as a biomarker of exposure, JUUL resulted in significantly higher levels of serum NE and MMP1 than UBC. Nicotine has previously been shown to activate neutrophils and macrophages to stimulate protease release, suggesting that nicotine is a common constituent amongst these three devices that induces protease release^25^. Propylene glycol (PG), vegetable glycerin (VG) and certain flavors are present in e-cigarettes and are likely not seen in combustible tobacco or heated tobacco products. Previous studies, including those from our group, demonstrated that unique components of e-cigarettes including PG/VG can be toxic, and may also induce protease release^57,58^. Thus, we speculate that despite producing fewer toxic chemicals than combustible tobacco products, unique chemicals in e-cigarettes may stimulate additional protease release beyond what is seen in combustible cigarettes or IQOS. Further, the association between JUUL use and the highest levels of systemic protease production after adjustment for serum nicotine may reflect the unique pharmacology of nicotine salts used in JUUL. Nicotine salts permit higher nicotine concentrations and greater bioavailability than free-base formulations, amplifying nicotine’s capacity to stimulate neutrophil protease release^59,60^. Hence, further studies are required to understand the potential long-term health consequences of e-cigarettes, especially the unique changes wrought by this class of ENDS.

A key strength of our study was its randomized crossover design, which by its nature, increases statistical power, reduces inter-individual variability, and provides more precise estimates of treatment effects. Another strength of our study was that we had a good representation of both sexes as well as non-Hispanic Black/African American and White participants, increasing the generalizability of our findings. We chose to expose chronic smokers to UBC, IQOS and JUUL since it would be unethical to expose vapers to (potentially more harmful) combustible cigarette smoke. However, a possible limitation of our study is that our participants were exclusively chronic cigarette smokers, and we did not study changes in protease levels in vapers. Further, the majority of subjects in our study were > 40 years old. This is consistent with the national U.S. average age of smoking cessation of 40 years^61^, and indicates that our study is representative, in terms of age, of U.S. combustible cigarette smokers. However, additional studies with younger subjects will be required to generate a fully representative dataset. Similarly, future smoking cessation studies and efforts should also target young adults to give the best chance to prevent the development of cigarette smoke-associated disease. Another limitation of our study was its acute nature in a relatively small sample of n = 21 participants, and larger, long-term studies are still needed to draw more definitive conclusions. In a subset of participants, peak protease levels were lower than baseline despite smoking exposure. This finding may reflect several biological mechanisms including: 1) baseline protease levels were not necessarily zero and may be elevated due to subclinical inflammation or recent nicotine exposures; 2) the timing of our sampling may have missed the true protease peak, as protease release can occur outside the collection window or remain sequestered in the airway rather than detectable in serum; and 3) nicotine itself has immunomodulatory effects and can transiently suppress certain inflammatory outputs via the cholinergic anti-inflammatory pathway and variation in circulating neutrophil counts or rapid neutralization by inhibitors such as alpha-1 antitrypsin may also contribute to this observation^62–64^. Additional studies using more frequent and/or longer sampling protocols will be important to capture peak responses and clarify the temporal dynamics of protease release more accurately. Last, we adjusted for potential confounding due to nicotine dose by normalizing peak protease levels to peak serum nicotine for each participant and device. Nicotine bioavailability and metabolism vary substantially across devices, with cigarettes producing rapid absorption and peak plasma levels within minutes, while e-cigarettes show slower and more variable T_max_ (time to maximum concentration) values^65^. Since protease release is mediated by immune cell activation and may lag behind nicotine T_max_, peak protease levels do not necessarily coincide temporally with peak nicotine levels^66^. Nonetheless, dividing peak protease by peak nicotine provides a conservative means of accounting for between-device differences in systemic nicotine exposure, thereby reducing confounding from nicotine’s direct effects on protease activity and allowing for a clearer assessment of device-specific influences.

In conclusion, we have shown that vaping and heated tobacco products increase blood protease levels, suggesting that chronic use of these devices may lead to development of significant pathology over time (e.g. COPD) in a manner similar to chronic cigarette use. Additional research to fully quantify the long-term effects of combustible cigarette alternatives on extracellular matrix homeostasis is warranted. Regulators should examine protease levels (including NE and MMP1) to make informed decisions regarding relative harm and regulations.

## Supplementary Information

Supplementary Information.

## Data Availability

Raw and analyzed data from this study will be made available upon request. Inquiries regarding data collection and analysis should be directed to the corresponding author.
