# Supplementary material for: The effect of nicotine delivery system on blood protease levels: a randomized crossover study
Source: Sci Rep. 2025 Oct 14;15:35864. doi: 10.1038/s41598-025-19832-8 (PMC12521394; doi:10.1038/s41598-025-19832-8)
Supplement: Supplementary file 1 — Supplementary Information. [file 41598_2025_19832_MOESM1_ESM.pdf]

The Effect of Nicotine Delivery System on Blood Protease Levels: A Randomized Crossover Study

Ava C. Wilson, PhD[1,2], Eleanor L.S. Leavens, PhD[3], Obdulia Covarrubias-Zambrano, PhD[4], Leah Lambart, PhD[3], Stefan H. Bossmann, PhD[4], Nicole L. Nollen, PhD[3] and *Robert Tarran, PhD[1]

Affiliations: 1. Division of Genetic, Environmental, and Inhalational Disease, Department of Internal Medicine, University of Kansas School of Medicine, Kansas City, KS, USA; 2. Division of Pulmonary, Critical Care, and Sleep Medicine, Department of Internal Medicine, University of Kansas School of Medicine, Kansas City, KS, USA; 3. Department of Population Health, University of Kansas School of Medicine, Kansas City, KS, USA; 4. Department of Cancer Biology, University of Kansas Comprehensive Cancer Center, University of Kansas School of Medicine, Kansas City, Kansas City, KS, USA.

*Correspondence to: Robert Tarran, PhD
Address: Division of Genetic, Environmental and Inhalational Disease
Department of Internal Medicine 1034 Lied Building Kansas University Medical Center, Kansas City, KC, 64160, USA. Email: rtarran@kumc.edu

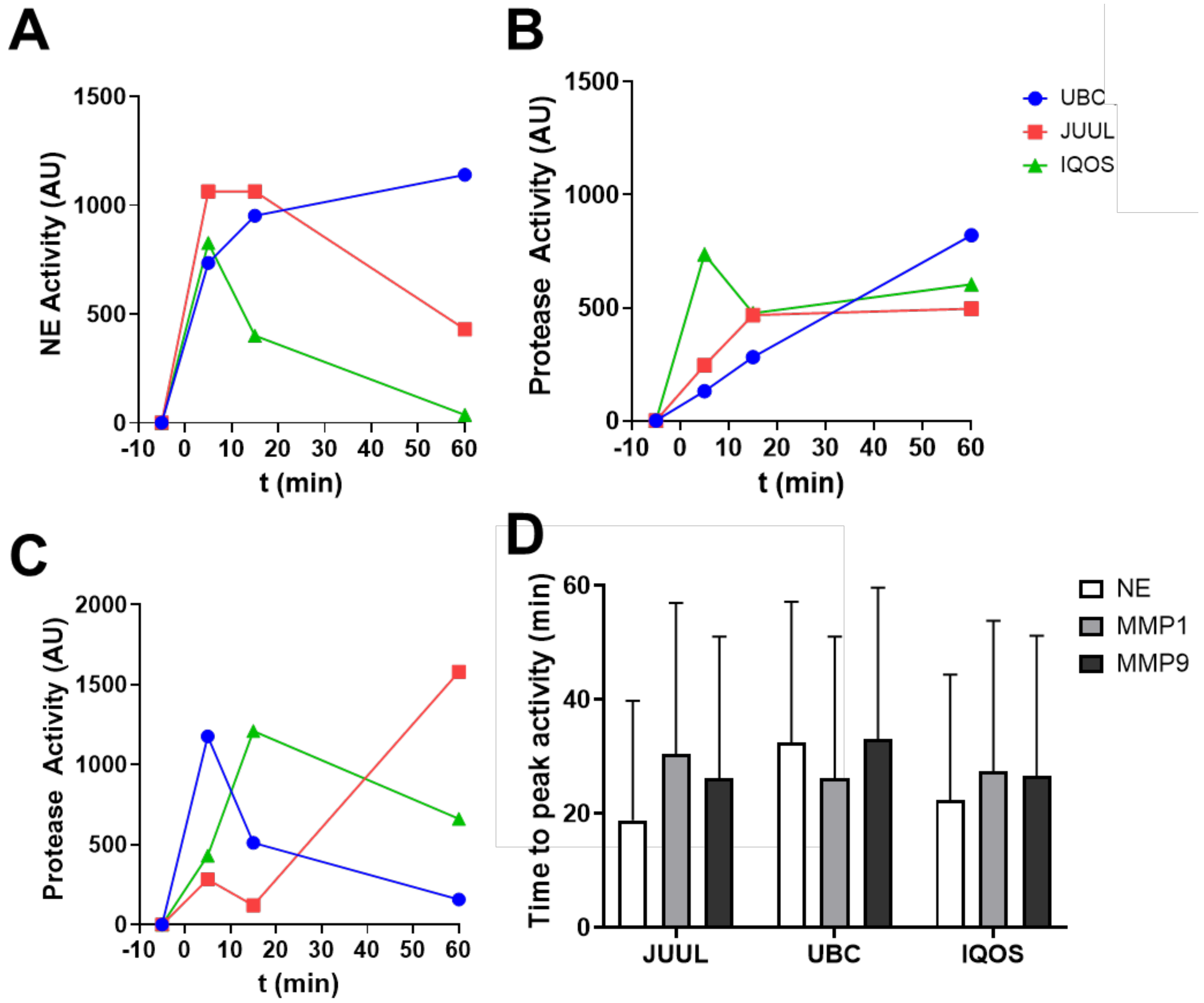

Figure E1. Representative raw data for NE, MMP1 and MMP9 across all devices. (*A*-*C*), Typical data traces for NE, MMP1 and MMP9 respectively, stratified by device type. (*D*) Bar graph showing time to peak protease activity stratified by protease and device type (all n=21).

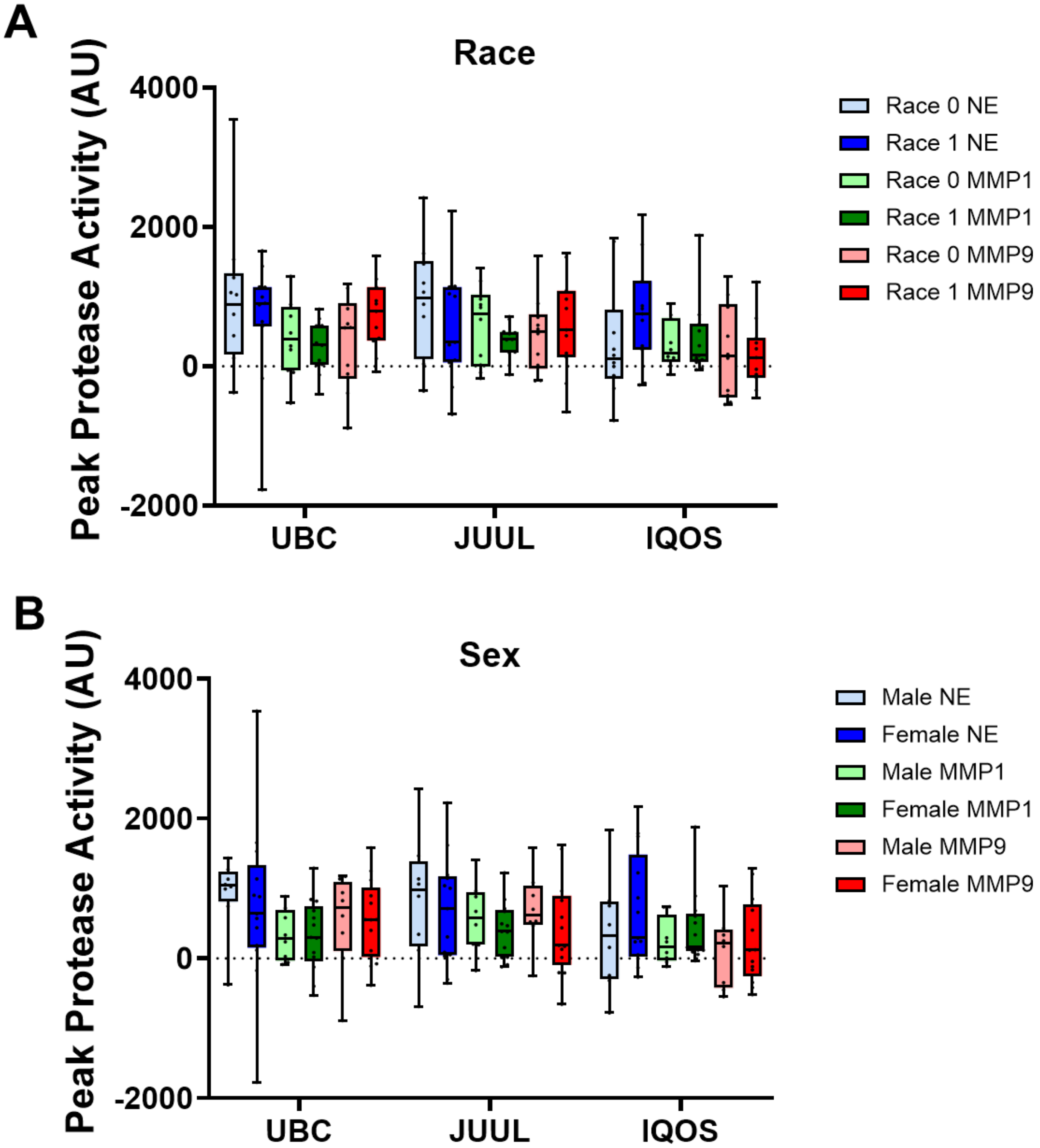

Figure E2. Increases in serum protease levels are independent of race and sex. (*A*) Bar graph showing peak protease levels stratified by race and device (all n=21). (*B*) Bar graph showing peak protease levels stratified by sex, and device (all n=21).

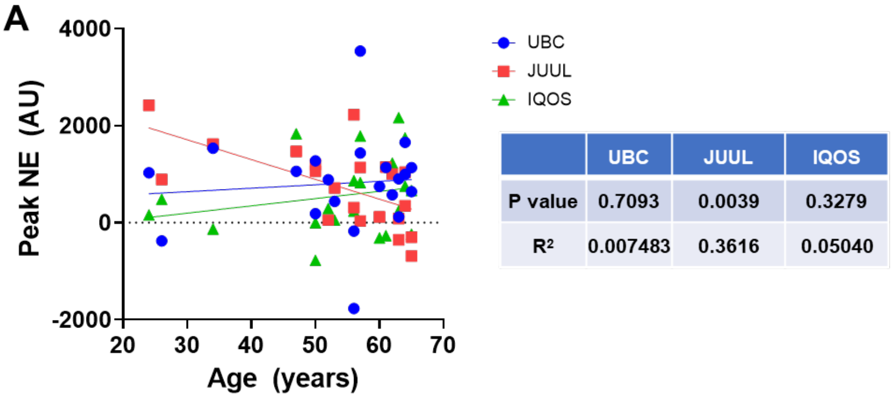

	UBC	JUUL	IQOS
P value	0.7093	0.0039	0.3279
R^2	0.007483	0.3616	0.05040

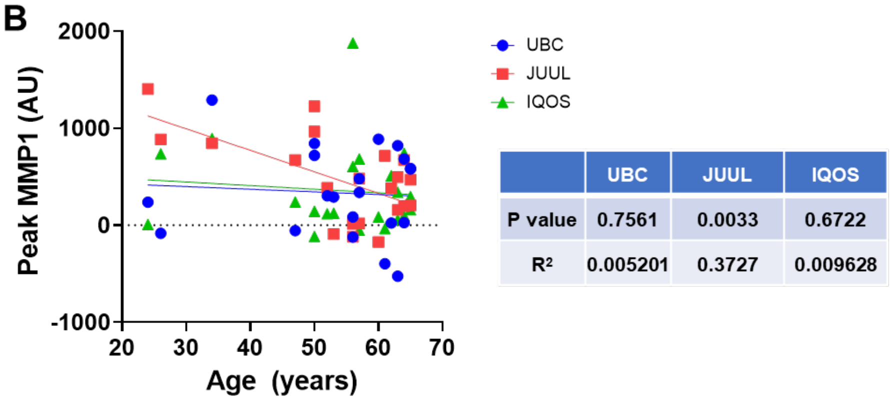

	UBC	JUUL	IQOS
P value	0.7561	0.0033	0.6722
R^2	0.005201	0.3727	0.009628

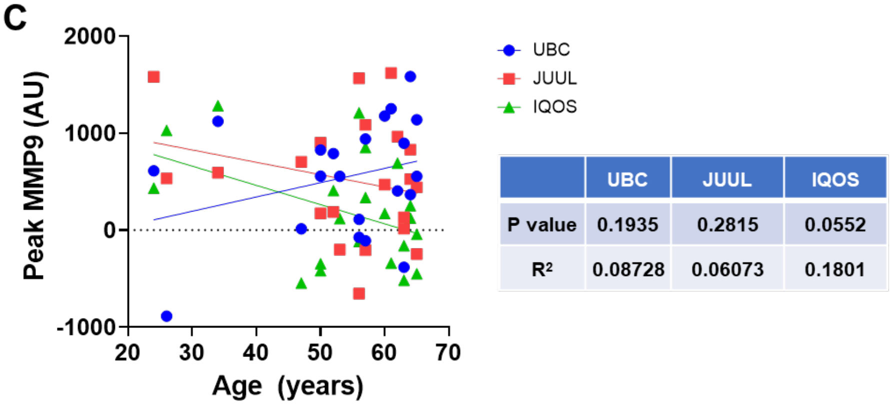

	UBC	JUUL	IQOS
P value	0.1935	0.2815	0.0552
R^2	0.08728	0.06073	0.1801

Figure E3. Peak protease levels generally do not correlate with subject age. Scatter plots of peak protease activity vs age stratified by device type for (*A*) NE; (*B*) MMP1 and (*C*) MMP9. All n=21 subjects.

Figure S4

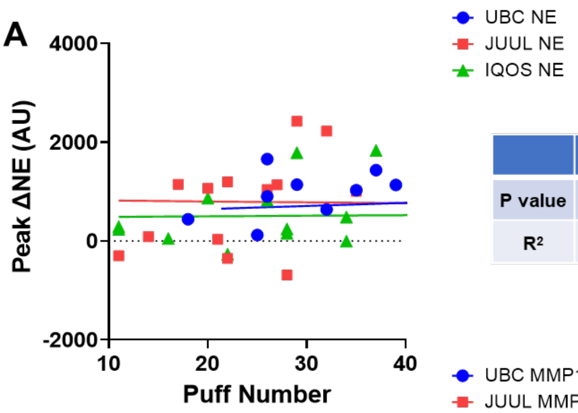

	UBC	JUUL	IQOS
P value	0.5780	0.5477	0.6247
R^2	0.01658	0.01934	0.01285

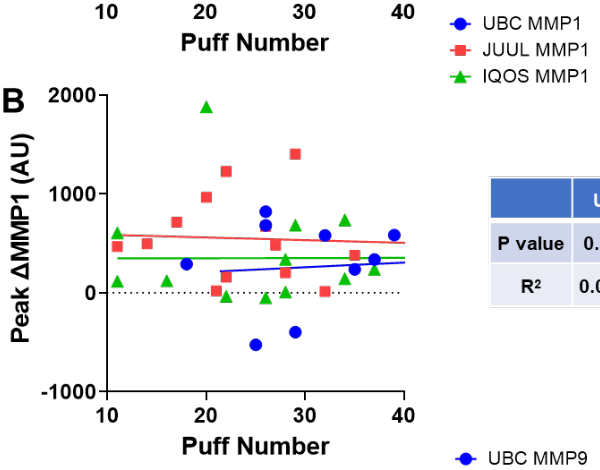

	UBC	JUUL	IQOS
P value	0.3541	0.0760	0.8970
R^2	0.04534	0.1563	0.0009058

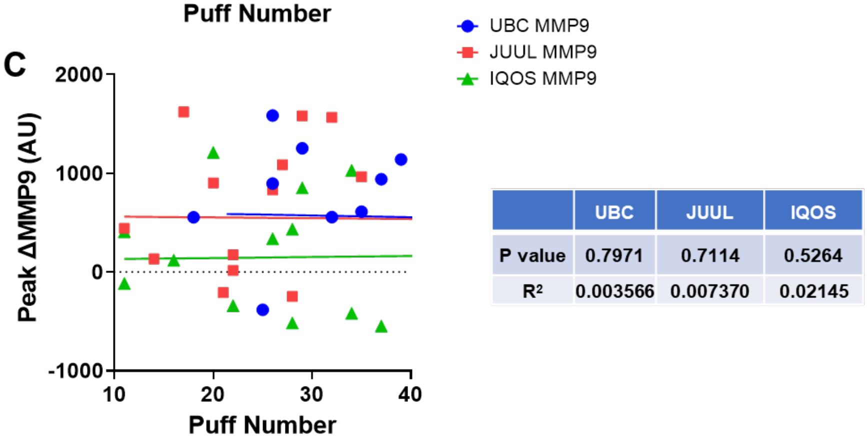

	UBC	JUUL	IQOS
P value	0.7971	0.7114	0.5264
R^2	0.003566	0.007370	0.02145

Figure S4. Peak protease levels do not correlate with number of puffs per session. Scatter plots of peak protease activity vs puff number per session, stratified by device type for (*A*) NE; (*B*) MMP1 and C, MMP9. All n=21 subjects.
